# Inverse ISAsomes in Bio-Compatible Oils—Exploring Formulations in Squalane, Triolein and Olive Oil

**DOI:** 10.3390/nano12071133

**Published:** 2022-03-29

**Authors:** Florian Trummer, Otto Glatter, Angela Chemelli

**Affiliations:** Institute of Inorganic Chemistry, Faculty of Technical Chemistry, Chemical and Process Engineering, Biotechnology, Graz University of Technology, Stremayrgasse 9, 8010 Graz, Austria; florian.trummer@uibk.ac.at (F.T.); otto.glatter@tugraz.at (O.G.)

**Keywords:** ISAsomes, inverse cubosomes, inverse hexosomes, self-assembly, nanostructures

## Abstract

In contrast to their more common counterparts in aqueous solutions, inverse ISAsomes (internally self-assembled somes/particles) are formulated as kinetically stabilised dispersions of hydrophilic, lyotropic liquid-crystalline (LC) phases in non-polar oils. This contribution reports on their formation in bio-compatible oils. We found that it is possible to create inverse hexosomes, inverse micellar cubosomes (Fd3m) and an inverse emulsified microemulsion (EME) in excess squalane with a polyethylene glycol alkyl ether as the primary surfactant forming the LC phase and to stabilise them with hydrophobised silica nanoparticles. Furthermore, an emulsified $L_{1}$-phase and inverse hexosomes were formed in excess triolein with the triblock-copolymer Pluronic^®^ P94 as the primary surfactant. Stabilisation was achieved with a molecular stabiliser of type polyethylene glycol (PEG)-dipolyhydroxystearate. For the inverse hexosomes in triolein, the possibility of a formulation without any additional stabiliser was explored. It was found that a sufficiently strong stabilisation effect was created by the primary surfactant alone. Finally, triolein was replaced with olive oil which also led to the successful formation of inverse hexosomes. As far as we know, there exists no previous contribution about inverse ISAsomes in complex oils such as triolein or plant oils, and the existence of stabiliser-free (i.e., self-stabilising) inverse hexosomes has also not been reported until now.

## 1. Introduction

Upon increased addition of amphiphilic molecules to an aqueous solution, hydrophobic forces will lead to the formation of aggregates that are known as micelles—the concentration threshold where micelles start to form is referred to as the critical micelle concentration [1,2,3,4,5]. When the concentration of amphiphilic molecules is further increased, the micelles will usually arrange into ordered structures and form lyotropic liquid-crystalline (LC) phases. Amphiphilic molecules, or surfactants, can be classified as ionic or non-ionic, and both types have advantages in certain fields of application [6]. Non-ionic surfactants comprise monoglycerides [7,8], fatty acids [9], phospholipids [10], amphiphilic block-copolymers [11,12,13,14,15,16] and many other classes of molecules [17].

### 1.1. ISAsomes–Definition and Nomenclature

Self-assembled LC-phases that are dilutable with an oil phase are usually termed as lipophilic and “inverse”—a term that is derived from the definition of inverse micelles. They comprise the following: inverse bicontinuous cubic phases ($V_{2}$), inverse hexagonal phases ($H_{2}$), inverse micellar cubic phases ($I_{2}$) and inverse micelles/microemulsions ($L_{2}$). The subscript 2 denotes that these LC-phases are lipophilic and “inverse”. If lipophilic LC-phases are capable of coexisting with a hydrophilic excess medium (typically water), they can be dispersed and stabilised in said medium, resulting in internally self-assembled, nanostructured particles [18,19,20,21,22,23,24,25,26] and are labelled according to the contained nanostructures mentioned above: bicontinuous cubosomes, hexosomes, micellar cubosomes and emulsified microemulsion (EME)) [25,27,28,29]. The whole family of particles is usually denoted as ISAsomes (internally self-assembled somes/particles) [30]. Due to historic reasons, the existing nomenclature is rather confusing, as “conventional” ISAsomes contain inverse LC-phases, a fact that will be emphasised again in the subsequent definition of the titular “inverse” ISAsomes. For this reason, the authors strongly recommend the use of categorisation “lipophilic/hydrophilic” for LC-phases in order to avoid confusion in this matter. Dispersed lamellar LC-phases form vesicles that are not included in the ISAsome family and are investigated separately [31,32,33]. For the sake of completeness, it is necessary to mention that amphiphilic molecules may also arrange into aperiodic quasicrystalline lattices and the production of these structures in thermodynamic equilibrium is a nontrivial task [34,35]. To the best of our knowledge, there exist no reports about dispersed quasicrystalline LC-phases that would be a new addition to the ISAsome family.

### 1.2. Stabilisation Schemes

The dispersed particles have to be stabilised kinetically in a solution with the help of a secondary emulsifier/stabiliser. ISAsomes are usually formulated as oil-continuous particles that are dispersed in an aqueous solution. Their stabilisation is usually achieved with the help of hydrophilic triblock co-polymers (PEO_n_PPO_m_PEO_k_) with high molecular weight (also known as poloxamers) and a high hydrophilic-lipophilic balance (HLB) [18,36,37]. Regardless of their hydrophilic character, these stabiliser molecules can interact with the dispersed LC-particles and lead to a change of the internal structure [38,39,40,41,42,43]. Other known concepts of emulsion stabilisation include the use of polyelectrolyte complexes [44], but there exist no reports about ISAsome stabilisation with this approach. Another popular technique for emulsion/dispersion stabilisation is the formation of Pickering or Ramsden emulsions, where the stabilisation of dispersed droplets is achieved with solid particles that include silica particles, laponite clay particles, latex particles and many others [45,46,47,48,49,50,51,52,53,54,55,56,57,58,59]. The stabilisation of emulsion/dispersion droplets strongly depends on the hydrophobicity of the solid stabiliser and the phase which is the best solvent for the emulsifying particles will usually be favoured as the external phase [45].

In recent years, more complex Pickering concepts have been introduced by various contributors. Instead of simply adsorbing spherical Pickering particles onto the surface of the emulsified droplets, stabilisation can be achieved by more complex mechanisms such as particle networks, rod-like particles, plate-shaped particles, etc., [60,61,62]. Food-grade Pickering emulsifiers are of special interest for bio-compatible applications, and various contributions have shown the emulsifying properties of particles based on calcium carbonate, cellulose nanocrystals/nanofibers and others [61,63,64,65,66,67]. In general, W/O (water-in-oil) Pickering emulsions have not been studied as thoroughly as their O/W (oil-in-water) counterpart [68,69,70,71,72,73]. As of now, no plant-based W/O Pickering emulsifier capable of producing droplet sizes <10 µm is known to the scientific community [60].

### 1.3. Applications

Due to their internal structure and large interfacial area, ISAsomes are unique carrier and delivery systems for a variety of hydrophilic, hydrophobic and amphiphilic molecules. Past contributions have shown their ability to encapsulate medical drugs that are used in the treatment of various types of cancer, neurodegenerative diseases and other sufferings [29,74,75,76,77,78,79,80,81,82,83,84,85,86,87,88,89,90,91,92,93]. The dispersed LC can be chosen according to the specific needs of the delivery application and phase transitions can be triggered by changes in pH [89,94,95,96,97,98,99,100], light stimulation [101], temperature changes [102], magnetic fields [103] and other external stimuli. In the case of a magnetic trigger, superparamagnetic iron oxide particles are incorporated into the lyotropic LC, and the phase transition can be attributed to the indirect heating of the “magnetocubosomes” by an alternating external magnetic field. In all cases, the phase transition properties may be used for a triggered release of drugs. In the field of nutritional science, ISAsomes can be used for the encapsulation of nutraceuticals such as natural phenolic antioxidants, curcumin, aromas, catechin and others [104,105,106,107,108,109,110,111,112,113,114,115,116,117,118,119,120,121]. The pH sensitivity of ISAsomes is of special interest here, as the gastrointestinal tract shows variations of the pH that enable a controlled release of nutrients where they are optimally digested [122,123]. In addition to oral drug delivery, the applicability of ISAsomes or similar systems in transdermal drug delivery has been shown [124,125,126]. Apart from specific applications, the formation of Lyotropic LCs and ISAsome-like structures during digestion processes are of special interest for nutritional science and biology and help to facilitate a better understanding of the human body [104,105,106,107,127] and understanding the processes behind the formation and interaction of these systems is a key element to unlock new applications in return [128,129].

### 1.4. Definition of Inverse ISAsomes and Research Goals

Until now, only “conventional” ISAsomes have been discussed. As stated, they are kinetically stabilised oil-continuous particles with a confined lipophilic lyotropic LC-phase or microemulsion. This type of system has been investigated in many contributions, and a variety of system compositions is known. Until recently, no reports about an inverse system had been made. In 2018, a reversion of the system was achieved by dispersing a hydrophilic LC with hexagonal nanostructure (*H*_1_-phase) in an alkane, resulting in the first report of inverse hexosomes [130]. This feat was achieved with a polyethylene glycol alkyl ether and a poloxamer as surfactants and the stabilisation of the dispersion was performed with a PEG-dipolyhydroxystearate with low HLB on one hand and hydrophobised silica nanoparticles on the other. Due to the system composition in excess tetradecane, the applicability of this system in bio-compatible applications is strongly limited, and to the best of our knowledge, no reports about other types of inverse ISAsomes existed until now. Alternative formulations in cosmetics-grade or food-grade oils would allow the development of delivery applications in an oil-continuous environment.

In this contribution, we demonstrate the formulation of inverse ISAsomes in three bio-compatible types of oil with increasing structural complexity. As stated above, inverse ISAsomes are defined as kinetically stabilised dispersions of hydrophilic LC-phases in an excess oil phase. Hydrophilic LC-phases are dilutable with water and comprise: bicontinuous cubic phases ($V_{1}$), hexagonal phases ($H_{1}$), micellar cubic phases ($I_{1}$) and micelles/microemulsions ($L_{1}$). To the best of our knowledge, this contribution is the first to report on the existence of inverse micellar cubosomes, an inverse emulsified microemulsions (EME) and an emulsified $L_{1}$-phase, thus adding three new members to the ISAsome family and enabling further research on the development of applications where the biocompatibility of all ingredients is of utmost importance. For our study, we chose three different oils as excess phase for inverse ISAsomes: squalane, a hydrocarbon that is often used in cosmetics, and triolein, a food-grade triglyceride that can be viewed as a model oil for natural plant oils. The third oil used was commercially available olive oil. As a first step, binary mixtures of a surfactant and water were produced and the appearing hydrophilic LC-phases were characterised with the help of small-angle X-ray scattering (SAXS). Polarisation light microscopy (PLM) was used as an additional tool in cases where SAXS measurements were not sufficient. As a next step, samples at promising surfactant concentration were loaded with increasing amounts of oil to test the compatibility of the LC-phases with the excess phase. The final step was the dispersion of the hydrophilic LCs in excess oil and the stabilisation of the droplets.

Our work was planned and conducted with the intent of providing a first “proof of principle” for the creation of inverse ISAsomes in (bio-)oil phases. For their characterisation, we relied on available in-house techniques. The particle-internal structure was determined with SAXS (or PLM, where needed) and the mean particle size was determined with dynamic light scattering (DLS) or PLM.

## 2. Materials and Methods

The aim of this contribution was the development of dispersion and stabilisation of hydrophilic lyotropic LC-phases in two naturals oil as the excess phase. While the LC-phase was formed by water and a primary surfactant, the stabilisation in excess oil was achieved with the help of a secondary surfactant or Pickering stabilising nanoparticles. Based on another contribution [130], a polyethylene glycol alkyl ether (C_i_E_j_) and a poloxamer were chosen as primary surfactants. Due to financial restrictions and for better reproducibility in possible future applications, technical-grade chemicals were used.

### 2.1. Materials

**Figure 1:**
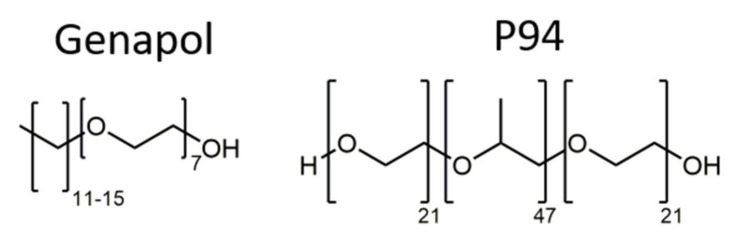
Chemical structures of the primary surfactants Genapol and P94.

**Figure 2:**
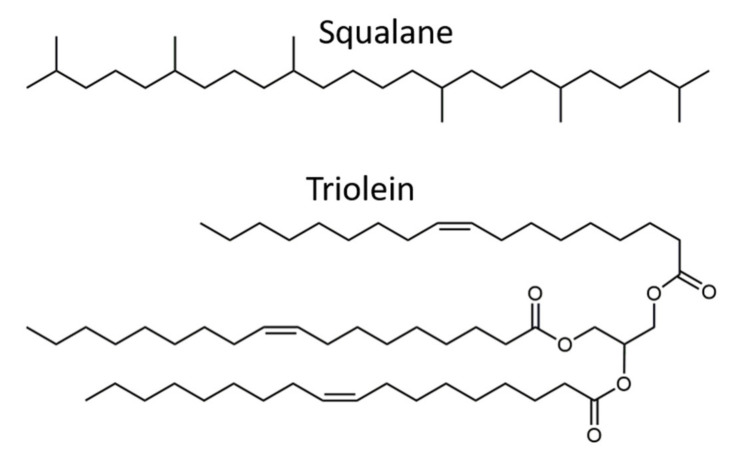
Chemical structures of the oils squalane and triolein.

Genapol LA 070 (Clariant AG, Bruckmühl, Germany) was the first primary surfactant used in this study. The surfactant molecules consisted of an alkyl chain with a length of 12–16 carbon atoms and seven ethylene oxide units. Pluronic^®^ PE9400 (in the following referred to as P94) (BASF SE, Ludwigshafen, Germany) was also used for the formation of the hydrophilic LC-phases. P94 is a triblock-copolymer (type A-B-A) consisting of a hydrophobic polyoxypropylene core (47 units) that is flanked by two hydrophilic polyoxyethylene blocks (21 units each). The chemical structures of both surfactants are shown in Figure 1. For the excess oil phase, three different oils were chosen: squalane (96% purity; Sigma-Aldrich Corp., Burlington, MA, USA) is a hydrocarbon often used in cosmetics. Recent studies have explored its applicability for transdermal delivery applications [126]. Triolein (90% purity; abcr GmbH, Karlsruhe, Germany) is a food-grade triglyceride found in many plant-based natural oils. It was used as a highly purified model oil to investigate the possibility to formulate inverse ISAsomes in commercially available food-grade oils (olive oil, etc.). Commercially available olive oil was the third oil used in this study. The chemical structures of triolein and squalane are shown in Figure 2.

**Figure 3:**
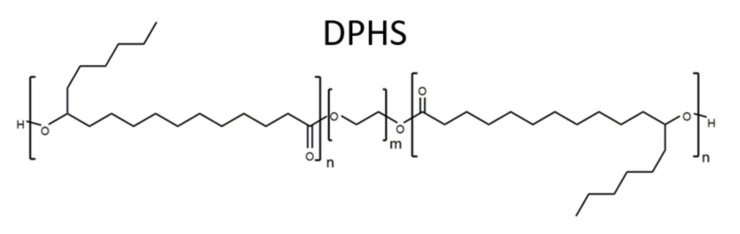
Chemical structure of Cithrol DPHS (m=30, n=9).

PEG-30 dipolyhydroxystearate (Cithrol DPHS from Croda International plc, Snaith, UK) was used as molecular stabiliser based on reports about its capability to form stable W/O emulsions in triolein and other oils and contributions that centre around its applicability for oral drug delivery [131,132]. Its chemical structure is shown in Figure 3. Hydrophobised silica nanoparticles of type Aerosil R711 (Evonik Industries AG, Essen, Germany) were used as Pickering- stabilisers for the formation of dispersions. Deionised water was used for all experiments.

### 2.2. Preparation Methods

Bulk samples typically were a binary system which consisted of distilled water and a primary surfactant (PS). By the addition of oil, a ternary system was formed. Bulk samples had a typical total mass of (1000 ± 20) mg. The ingredients were weighed with an analytical balance (∆*m* = ±0.1 mg) and stored in 4 mL vials and heated up to a temperature of 80 °C for 5 min in order to melt any occurring LC-phases before agitation with a vortex (RS-VA 10 by Phoenix Instrument, Garbsen, Germany) until room temperature was reached. The binary system water/surfactant is characterised by the mass ratio of the primary surfactant (PS)

(1) $$\delta =\frac{m_{PS}}{m_{PS}+m_{H_{2}O}}\cdot 100$$

Ternary systems were formed by adding defined amounts of oil (given in %_w_ of the total bulk mass) to the binary system. After vortexing, bulk samples with the PS Genapol were centrifuged for one hour at 4500 rpm (Labofuge 400R by Heraeus, Hanau, Germany) in order to remove entrapped air bubbles that had formed during vortexing. Samples with the primary surfactant P94 did not require this procedure due to the low-foaming characteristics of this surfactant. All bulk samples were equilibrated at room temperature for one day before any measurements were taken.

Dispersions consisted of the components of the ternary system along with an additional surfactant (stabiliser). The stabiliser was dissolved in the oil phase (0.5%_w_-solution of Pickering stabiliser; 1%_w_-solution of molecular stabiliser). An ultra-sonication device (Vibra-Cell by Sonics & Materials, Newtown, CT, USA) with a tapered tip was used at a power of 120 W in pulsed mode (0.3 s pulses with breaks of 2 s). The samples were placed in a water bath with additional ice to avoid over-heating of the components. As a first step, the PS was combined with the oil and the stabiliser-solution and dispersed for 2 min before the last component, distilled water, was added and the samples were again dispersed for 10 min. The dispersions can be characterised by the δ-value and two more variables related to the amount of stabiliser and the total concentration of ISAsomes in the dispersion:

(2) $$\beta =\frac{m_{Stabiliser}}{m_{PS}+m_{H_{2}O}}\cdot 100$$

(3) $$\phi =\frac{m_{PS}+m_{H_{2}O}}{m_{PS}+m_{H_{2}O}+m_{Stabiliser}+m_{Oil}}\cdot 100$$

Dispersion samples had a typical total mass of (2000 ± 50) mg and were stored in 4 mL vials. Unless specified otherwise, all dispersion samples were equilibrated at T_RT_ for 1 h before further characterisation with SAXS, dynamic light scattering (DLS) or polarisation light microscopy (PLM).

**Polarisation light microscopy (PLM):** A Leica DM2500 M microscope (Leica Camera AG, Wetzlar, Germany) with two polarisation filters was used for the determination of anisotropic samples (refer to chap. 2.4 for details). The samples were squeezed between two glass plates and investigated in transmittant light mode with various objective lenses. A Sony DXC-390P camera (Sony, Tokio, Japan) was mounted on the microscope and the pictures were recorded with the software Sarfusoft 2.1 (Nanolane, Le Mans, France).

**Dynamic light scattering (DLS):** The mean hydrodynamic radius of the dispersion droplets was determined with dynamic light scattering (DLS). The equipment consisted of a Litesizer^TM^ 500 (Anton Paar GmbH, Graz, Austria) that operates with a solid-state semiconductor laser diode with a wavelength of 658 nm at a power of 40 mW. The light scattering signal of each sample was measured five times for 1 min at a scattering angle of 90° and a temperature of 25 °C. The data was analysed with the software Kalliope 2.20.2 by the application of the cumulant method [133]. An Abbemat 550 refractometer and an MCR 502 rheometer (rotational mode) (both from Anton Paar GmbH, Graz, Austria) were used for the determination of the index of refraction and the viscosity of oils at 25 °C.

**Small-angle X-ray scattering (SAXS):** A high flux SAXSess camera (Anton Paar GmbH, Graz, Austria) connected to a Debyeflex 3003 X-ray generator (Ge-Electric, Frankfurt, Germany) with a sealed-tube Cu-anode was used. The operating settings of the generator were set to 40 kV and 50 mA which results in the X-ray wavelength *λ* = 1.5420 Å (Cu-K-α radiation). A line-shaped X-ray beam (17 mm horizontal extension at the sample) was focused with a Goebel mirror and collimated with a Kratky slit. The scattered beam was measured in transmission mode with a 1D MYTHEN-1k microstrip solid state detector (Dectris AG, Baden, Switzerland) in a range of *q* = (0.1–6) nm^−1^, where *q* denotes the absolute value of the scattering vector defined as $q=\frac{4\pi }{\lambda }\cdot \sin\left(\frac{\theta }{2}\right)$ with the total scattering angle *θ*. In special cases where a higher resolution was needed, a SAXSpoint 2.0 system (Anton Paar GmbH, Graz, Austria) was used. The camera was connected to a Primux 100 micro X-ray source with an X-ray wavelength *λ* = 1.5418 Å (Cu-K-α radiation) and a 2D Eiger detector was used. The measurements were taken in a range of *q* = (0.0012–5.5) nm^−1^, and the data acquisition and conversion were performed with the software packages SAXSdrive (version 2) and SAXSanalysis (version 4). Unless specified otherwise, all measurements were taken at a temperature of 20 °C (T_RT_).

## 3. Results

### 3.1. Investigations on Bulk Samples

As stated in the section “Materials and Methods”, binary bulk samples are formed by mixing the primary surfactant (PS) with water. A ternary bulk phase was formed by addition of oil to the binary mixture to test the ability of the hydrophilic LC-phases to coexist with excess amounts of oil. The bulk samples were investigated with SAXS measurements and, where necessary, polarisation light microscopy (PLM) was used to investigate the structure of the LC-phases. Binary bulk phases were typically clear, highly viscous liquid crystals. Microemulsions/$L_{1}$-phases typically appeared as transparent fluids. Upon the addition of excess oil, a turbid, inhomogeneous two-phase system (oil-saturated LC + excess oil) was obtained.

#### 3.1.1. Genapol–Squalane System

**Figure 4:**
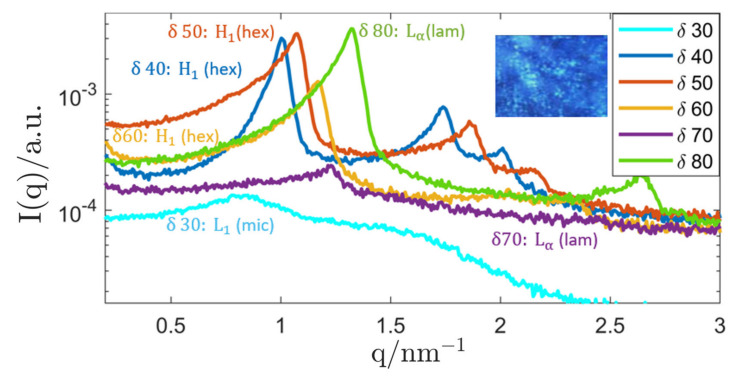
Selected SAXS measurements of samples with varying surfactant concentration *δ* in the range *δ* = (30–80). The hexagonal scattering curves are emphasised in the plot. For better readability, the curves are labelled with the assigned LC-phases $H_{1}$…hexagonal, $L_{\alpha }$…lamellar, $L_{1}$…isotropic micellar. The inset shows the PLM image (magnification 20) of a liquid-crystalline phase with *δ* = 70 that is typical for lamellar anisotropy (maltese crosses weakly visible). All graphs were shifted by an arbitrary factor for better readability.

The first system was based on the PS Genapol LA 070, a polyethylene glycol alkyl ether that has shown beneficial behaviour in past studies [130,134]. The SAXS curves of the binary system Genapol–water are shown in Figure 4. For surfactant concentrations 40≤δ≤60, a hexagonal phase ($H_{1}$) was formed that changed to a lamellar structure ($L_{\alpha }$) for δ≥70 (characterised by equidistant peak positions and the appearance of characteristic “maltese crosses” in the PLM image). The hexagonal phase was identified by the characteristic position of the SAXS-peaks ($q_{0},\sqrt{3}q_{0},2q_{0}$) and the hexagonal lattice parameter can be calculated from the position of the primary peak $q_{0}$ [7]. The lattice parameter changed from (7.19 ± 0.05) nm (δ=40) to (6.21 ± 0.04) nm (δ=60) due to a decreased hydration of the hydrophilic headgroups at a lower water content. At lower surfactant concentrations (δ≤30), the formation of an isotropic micellar solution ($L_{1}$) could be observed.

**Figure 5:**
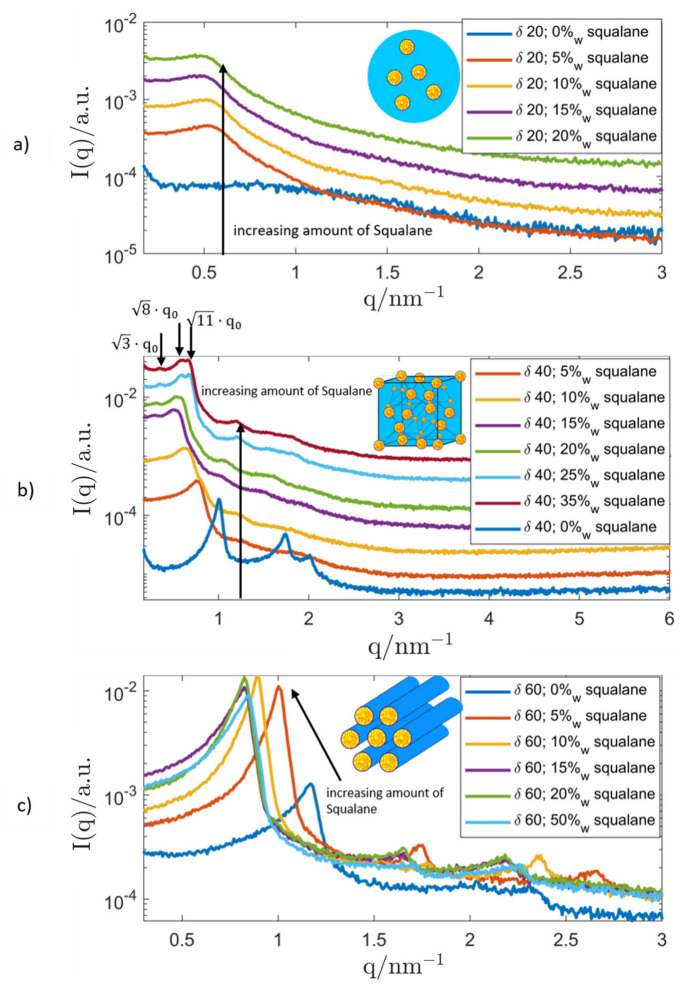
SAXS curves of samples with varying surfactant concentration: *δ* = (**a**) 20; (**b**) 40; (**c**) 60 and increasing amounts of squalane (given in %_w_). The downward arrows in the middle panel show the position of the characteristic SAXS-peaks of the micellar cubic Fd3m phase. All graphs were shifted by an arbitrary factor for better readability. The insets show schematic depictions of the detected LC-phases: Top: microemulsion; Middle: micellar cubic (*I*_1_, Fd3m); Bottom: hexagonal (*H*_1_).

Based on those results, bulk samples at selected surfactant concentrations were loaded with increasing amounts of squalane to investigate its influence on the LC-structure and the possibility of full oil-saturation while maintaining the LC-phase. The corresponding SAXS measurements are shown in Figure 5.

At low surfactant concentrations δ, a microemulsion (swollen micellar solution) was formed by incorporation of squalane into the micellar aggregates. The corresponding SAXS curves show the broad correlation peak that corresponds to the mean centre-to-centre distance of the micelles.

As can be seen, the system saturated at an oil concentration of 15%_w_ without deterioration of the structure. Therefore, the most important prerequisite for the dispersion of the system and the formation of an inverse emulsified microemulsion (EME) was given.

Based on the behaviour of the binary system Genapol/water, the oil-loading of a hexagonal structure was attempted. However, upon incorporation of squalane into its structure, bulk samples with a surfactant concentration δ=40 showed a radical change in phase behaviour, as revealed by the SAXS measurement: The initial hexagonal phase started to change upon addition of small amounts of squalane. At higher oil concentrations (≥10%_w_), a micellar cubic phase ($I_{1}$) with Fd3m symmetry started to develop. The structure of this LC-phase was first described by Mariani et al. [135]. As expected, the peaks shifted towards smaller *q*-values with increasing concentration of squalane which signifies a swelling of the structure, as more and more oil was incorporated into the micelles. When the oil concentration was increased even further, a micellar cubic phase with Fd3m symmetry started to coexist with the microemulsion until the pure, oil-saturated Fd3m-phase was found at an oil concentration of 25%_w_ and a lattice parameter of (31.1 ± 0.2) nm. Investigations with the polarised light microscope (PLM) showed no signs of anisotropy, which supported the existence of a cubic LC-phase. As the system could coexist with excess amounts of oil, the prerequisite for the dispersion and creation of inverse micellar cubosomes was given.

**Figure 6:**
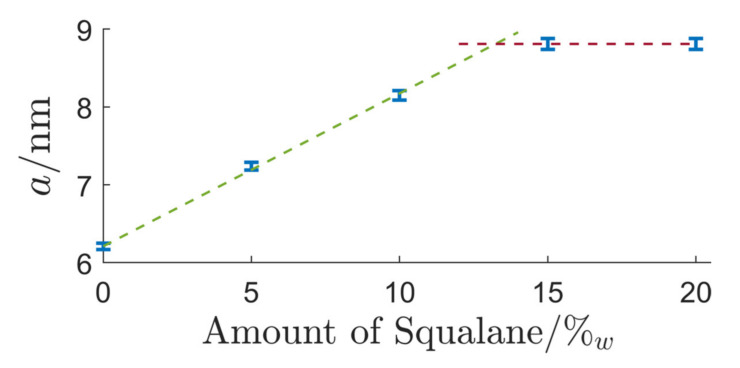
Depiction of the growing lattice parameter *a* of the hexagonal phase with addition of increasing amounts of squalane. A linear fit was applied to the growth region and the saturated regime to determine the saturation oil concentration.

At even higher values of the surfactant concentration (δ=60), the expected swelling of the hexagonal structure could be observed. The hexagonal LC-phase remained stable, and the lattice parameter of the binary mixture (6.21 ± 0.04) nm started to swell until saturation was reached at a squalane concentration of 13.2%_w_ and a lattice parameter of (8.81 ± 0.07) nm, as shown in Figure 6.

An overview of binary bulk phases observed at the surfactant concentrations δ=20,40,60 along with the corresponding, oil-saturated systems is given in Table 1.

As in the cases before, the stability of the hexagonal phase upon saturation with squalane was the most important prerequisite for the dispersion of the hydrophilic LC and the formation of inverse hexosomes.

The combination of the binary system Genapol/H_2_O with triolein as the excess oil phase showed no promising results in experiments. The addition of triolein greatly influenced the stability of the LC-phases and only less well-defined mixed-phase configurations could be achieved in the ternary system. Therefore, this system configuration was omitted for the production of inverse ISAsomes and an alternative primary surfactant, P94, was ultimately used in combination with triolein.

#### 3.1.2. P94/Triolein System

**Figure 7:**
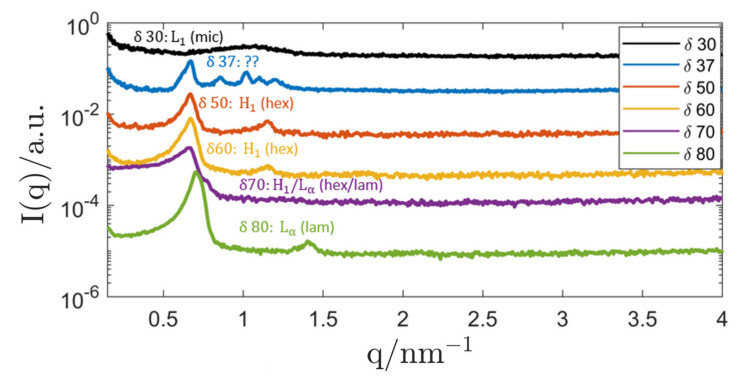
SAXS curves of the binary system P94/H_2_O with varying surfactant concentration *δ*. For better readability, the curves are labelled with the assigned LC-phases: $L_{1}$…isotropic micellar solution, $H_{1}$…hexagonal, $L_{\alpha }$…lamellar, $H_{1}/L_{\alpha }$…two-phase mixture hexagonal/lamellar. The hexagonal scattering curves are emphasised in the plot. The sample with *δ* = 37 was equilibrated at a temperature of 5 °C for 1 day and at $T_{RT}$ for another day. The measurements of samples with *δ* ≤ 37 were performed with the SAXS camera “SAXSpoint 2.0” for better resolution. All curves were shifted by an arbitrary factor for better readability.

In analogy to the Genapol-system, binary bulk samples with different ratios of water/P94 were produced. Selected SAXS curves are presented in Figure 7. The results were compared with a known phase diagram from another contribution [13], and for the most part, no discrepancies with literature could be identified. Starting at a surfactant concentration of δ=30, a micellar solution ($L_{1}$) was formed. However, the cubic phase ($I_{1}$) predicted by the phase diagram at a surfactant concentration of δ=37 could not be confirmed with SAXS measurements. The SAXS curve showed some unidentified type of order that could signify a mixed-phase system of $H_{1}$ and another phase with unidentified order. As no pure, non-mixed LC-structure could be confirmed with SAXS measurements, samples with this composition were omitted for the production of inverse ISAsomes. When raising the surfactant concentration to δ=50, the expected hexagonal ($H_{1}$) phase was formed and maintained a lattice parameter of (10.93 ± 0.11) nm until it changed to a mixed-phase system ($H_{1}+L_{\alpha }$) at δ=70 and to a lamellar structure at even higher values of δ.

**Figure 8:**
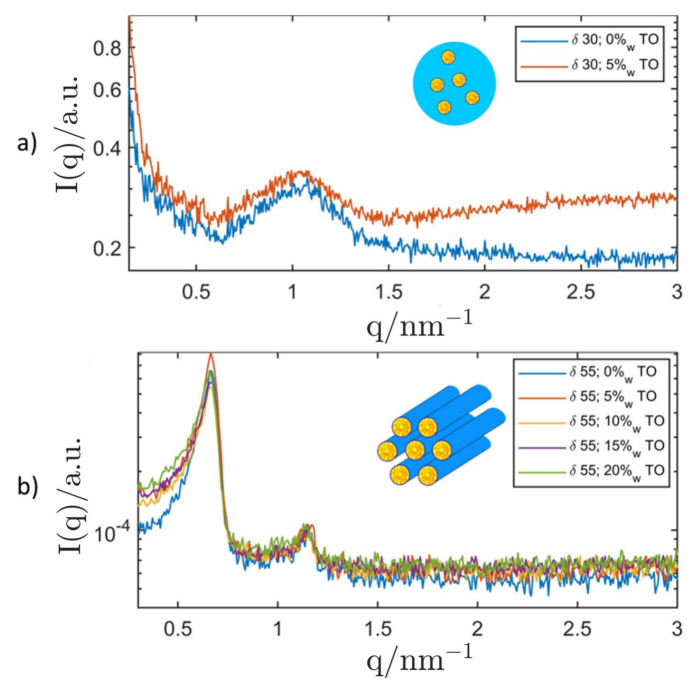
SAXS curves of the ternary system P94/H_2_O/TO at different surfactant concentrations (*δ* = (**a**) 30 & (**b**) 55) with added triolein (TO) and the corresponding binary systems without added oil. The measurements for the samples with δ=30 were performed with the SAXS camera "SAXSpoint 2.0” for better resolution. The insets show schematic depictions of detected LC-phases. Top: micellar isotropic *L*_1_-phase; Bottom: hexagonal *H*_1_-phase.

Again, samples at selected surfactant concentrations were loaded with increasing amounts of oil and the robustness of the LC-phases was verified with SAXS (shown in Figure 8). Triolein was chosen as the oil phase as it is a triglyceride with a well-defined molecular structure that can be used as a model oil for commercially available food-grade oils (e.g., olive oil). It is necessary to mention at this point that polarisation light microscopy (PLM) was hardly applicable in this system due to a low birefringence of the $H_{1}$ and $L_{\alpha }$ phases formed by the primary surfactant P94.

At a surfactant concentration of δ=30, the addition of triolein to the binary micellar solution showed no measurable effect on the micellar structure, as can be seen in Figure 8. The broad SAXS correlation peak (starting at q=1.5 nm^−1^) is characteristic for samples that contain a triglyceride and can be attributed to the molecular order in these oils [136]. It can be deduced, that micelles formed by the primary surfactant P94 incorporate hardly any triolein. Due to the robustness of the micellar structure against addition of excess amounts of triolein, the decision was made to attempt a dispersion of this system.

A surfactant concentration of δ=55 was chosen for oil-loading of the hexagonal structure: As for the other two structures, the hexagonal lattice seemed to incorporate hardly any oil and the prerequisite for the dispersion of the system in excess triolein was given. The oil compatibility of hydrophilic LC-phases formed by the primary surfactant P94 was also tested with squalane where a similar result was obtained. As hardly any oil is incorporated into the LC-structures, the ability to coexist with a large variety of oils could be given. An overview of binary bulk phases observed at the surfactant concentrations δ=30 and 55 along with the corresponding, oil-saturated systems is given in Table 2.

### 3.2. Dispersion and Stabilisation

As both systems, Genapol/H_2_O/squalane and P94/H_2_O/triolein showed capabilities to form stable hydrophilic LC-phases in the presence of excess amounts of oil, the dispersion and stabilisation of both systems was attempted. The creation of dispersion samples is described in the section “Materials and Methods”. ISAsome dispersions typically appear as a turbid medium with white colour. Samples in excess squalane had a white colour, while samples dispersed in triolein and olive oil appeared as yellow, milk-like liquid.

#### 3.2.1. Genapol/Squalane System

A variety of stabilisers was tried for dispersed LC-phases of this system. Stabilisation attempts with polymeric stabilisers (e.g., DPHS and reverse Pluronics) were not fruitful and all dispersion samples showed aggregation and phase separation rather quickly.

**Figure 9:**
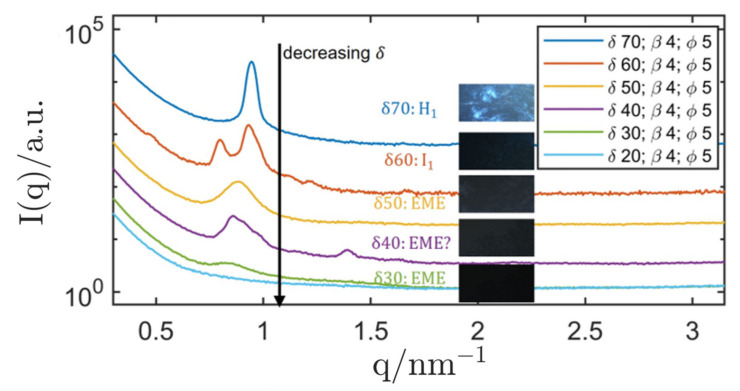
SAXS curves of R711 Pickering emulsions (Genapol/H_2_O in squalane) with β=4 and varying surfactant concentration δ. The inset shows the respective PLM images. The sample with δ=60 was heated to 37 °C before measuring at 20 °C to obtain the Fd3m structure. In order to improve the quality of the measurement data of this sample, a scan of the empty capillary was taken and subtracted. For better readability, the curves are labelled with the assigned LC-phases: $H_{1}$…hexagonal, EME…emulsified microemulsion $I_{1}$…micellar cubic (Fd3m symmetry). The SAXS curves of all samples were measured with the SAXS camera “SAXSpoint 2.0” for better resolution. All graphs were shifted by an arbitrary factor for better readability.

The final stabilisation attempt was performed with R711-nanoparticles. The impact of a varying surfactant concentration δ at a fixed stabiliser concentration β and a fixed ISAsome concentration ϕ was investigated. The results are displayed in Figure 9. In analogy to previous works [130], the primary hexagonal peak could be clearly measured in this Pickering emulsion, while higher-order peaks were often hard to detect due to the background scattering intensity of the silica nanoparticles. The presence of the hexagonal phase (δ=70) was confirmed with the help of the PLM that showed clear signs of non-lamellar anisotropy. The hexagonal lattice parameter was determined as (8.28 ± 0.07) nm, which was slightly lower than the value measured in the oil-saturated bulk phase. This phenomenon could be attributed to an interaction of the stabiliser with the LC-phase, where parts of the primary surfactant were adsorbed at the silica nanoparticles until they were saturated [130]. This also led to a shift of the surfactant concentration δ between bulk samples and corresponding dispersion that could be observed for all structures. Inverse hexosomes, e.g., were found at δ=70 while corresponding, oil-saturated hexagonal bulk samples were only found at a lower surfactant concentration $\left(\delta =60\right)$. Nevertheless, the production of Pickering-stabilised inverse hexosomes in excess squalane was successful.

The characterisation of the dispersion with δ=60 with SAXS revealed the existence of a micellar cubic phase with Fd3m symmetry. The structure only developed after heating the sample to 37 °C before finally taking the measurement at 20 °C, which could signify that the thermodynamical equilibrium of the LC-phase is only reached after applying this heating scheme. It might be related to the fact that samples were cooled in an ice bath during dispersion. After the described heating, the samples maintained their Fd3m structure when they were being stored at a temperature of 20 °C. A cooling of the sample to 5 °C induced a change to an inverse emulsified microemulsion (EME).

**Figure 10:**
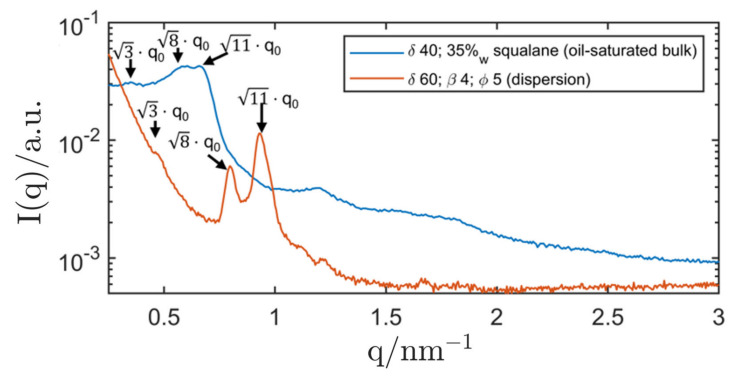
SAXS curves of R711 Pickering emulsions (Genapol/H_2_O in squalane) with a contained micellar cubic phase (Fd3m symmetry) and arrows signifying the position of the characteristic SAXS peaks of this phase. The dispersion was heated to 37 °C before measuring at 20 °C to obtain the Fd3m structure. For comparison, the corresponding oil-saturated bulk phases is also displayed. In order to improve the quality of the measurement data of the dispersion, a scan of the empty capillary was taken and subtracted. The SAXS curve of the dispersion was measured with the SAXS camera “SAXSpoint 2.0” for better resolution.

As can be seen in Figure 10, the micellar cubosome structure was clearly shifted with respect to the oil-saturated bulk phase. While the latter had a lattice parameter of (31.1 ± 0.2) nm, the cubosomes showed a smaller lattice parameter of (22.3 ± 0.2) nm. As mentioned above, the stabilisation of the cubosomes with silica nanoparticles might lead to a significant decrease in the effective surfactant concentration δ (also hinted at by the relatively large shift of δ when compared to the oil-saturated bulk-phase). SAXS measurements clearly showed the micellar cubic structure of the dispersion, and the PLM showed no signs of anisotropy. Therefore, the development of Pickering-stabilised inverse micellar cubosomes (Fd3m symmetry) was successful.

**Figure 11:**
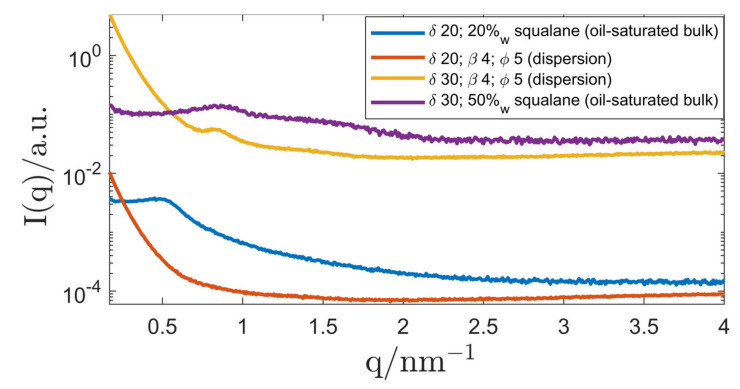
SAXS curves of R711 Pickering emulsions (Genapol/H_2_O in squalane) with $\delta =\left(20–30\right)$. For comparison, the corresponding oil-saturated bulk phases are also displayed. The SAXS curves of the dispersions were measured with the SAXS camera “SAXSpoint 2.0” for better resolution. All graphs were shifted by an arbitrary factor for better readability.

When the surfactant concentration δ was chosen below 60, the structure gradually changed to an inverse emulsified microemulsions (EME) and a dispersion with δ=40 showed signs of a mixed-phase system, signified by the appearance of a second peak and a disturbed primary peak. At δ=30, the SAXS measurement showed clear signs of an inverse emulsified microemulsion (EME). The dispersion was compared with SAXS measurements of oil-saturated bulk samples (microemulsions) and the results are shown in Figure 11.

At a surfactant concentration of δ=30, the peak position of the bulk sample (oil-saturated microemulsion) was in accordance with that of the corresponding dispersion. Therefore, the existence of an inverse emulsified microemulsion (EME) could be shown. When lowering δ to 20, the correlation peak of the EME could not be measured with SAXS anymore.

All Pickering emulsions showed aggregation nearly immediately which prevented the characterisation of the particle size with DLS. The aggregate size was measured with the polarisation light microscope (PLM) and was estimated to be in a range of (100–300) µm. No phase separation was observed in a time span of one month.

#### 3.2.2. P94/Triolein System

Based on knowledge gained from previous studies [132], DPHS was chosen for the steric stabilisation of inverse ISAsomes in triolein. Again, the surfactant concentration δ was varied and the appearing structures were characterised with SAXS. In addition, triolein was ultimately exchanged with commercially available olive oil and the effect on the inverse ISAsomes was observed. The broad SAXS correlation peak typically shown by triglycerides often covered the signal from the structure of dispersed LC-phases which sometimes led to the necessity of further data processing to reveal structures.

**Figure 12:**
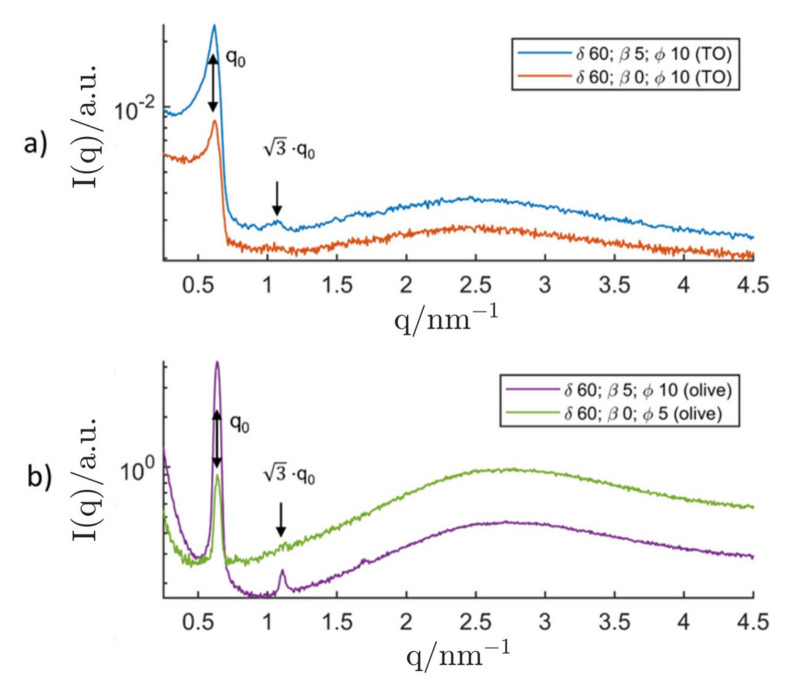
SAXS curves of successfully produced inverse hexosomes made of P94/H_2_O. The upper graph (**a**) shows the hexosomes in triolein (TO) with (β=5) and without (β=0) additional stabiliser DPHS and a constant ISAsome concentration ϕ=10. At the bottom (**b**), the analogous system in olive oil is displayed. The SAXS curves of the bottom graph were measured with the SAXS camera “SAXSpoint 2.0” for better resolution. All graphs were shifted by an arbitrary factor for better readability.

At a surfactant concentration of δ=60, inverse hexosomes with a lattice parameter of (11.75 ± 0.12) nm were formed in triolein. The stabilisation with DPHS led to a typical hydrodynamic diameter in the sub-µm regime (measured with DLS) and no phase separation could be observed over the course of a month. The DLS data for the inverse hexosomes can be found in Table 3. When the stabiliser was omitted (β=0), inverse hexosomes with an identical lattice parameter were formed and the dispersion showed surprisingly good steric stability. Aggregates with a size of (100–300) µm (measured with PLM) were formed and no phase separation could be observed. The stabilisation was most likely achieved by the long polymeric chains of the primary surfactant (P94) that ensure a minimum steric effect. When triolein was exchanged with commercially available olive oil, the inverse hexosomes were found to have a lattice parameter of (11.39 ± 0.10) nm which is slightly smaller than the value measured for the analogous system in triolein. It is probable that surface-active impurities in the olive oil are responsible for this small change of the lattice parameter. Stabilisation with DPHS showed mediocre results: The inverse hexosomes had a typical diameter of (50–100) µm (measured with PLM). The self-stabilising system without DPHS showed typical aggregate sizes of (100–300) µm (measured with PLM). The resulting SAXS curves of the inverse hexosomes are shown in Figure 12. The ISAsome concentration for the self-stabilising hexosomes in olive oil was lowered to ϕ=5 to achieve a better dispersion quality.

**Figure 13:**
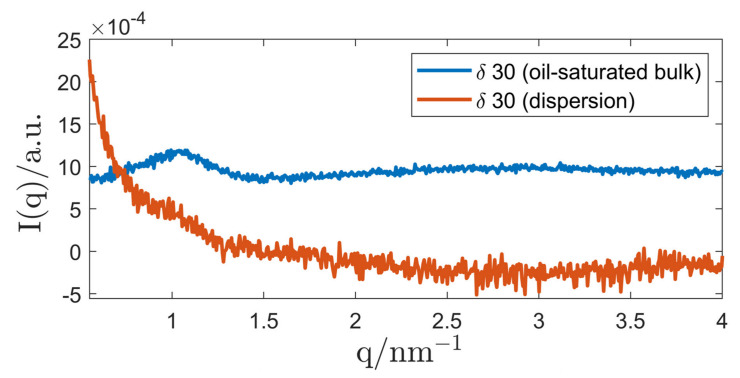
SAXS curves of an inverse EME made of P94/H_2_O in TO. For comparison, the oil-saturated bulk phase is also displayed. All graphs were shifted by an arbitrary factor for better readability. A scattering curve of pure TO was subtracted from the dispersion curve to reveal the correlation shoulder of the emulsified *L*_1_-phase. The measurements were performed with the SAXS camera “SAXSpoint 2.0” for better resolution.

For dispersions with a lower surfactant concentration of δ=30, SAXS curves showed a correlation shoulder at a position that is in accordance with the correlation peak of the oil-saturated bulk sample, as shown in Figure 13, and DLS measurements revealed a hydrodynamic diameter below 300 nm as shown in Table 3. As discussed above, micelles made of P94/H_2_O do not incorporate measurable amounts of oil, which makes the categorisation of the (emulsified) bulk phase as “(emulsified) inverse microemulsion” questionable. In lack of an existing nomenclature, we decided to name the dispersed system as “emulsified *L*_1_-phase”.

SAXS measurements of the corresponding system in olive oil did not reveal the existence of an inverse EME or an emulsified *L*_1_-phase. It is possible that the micellar structure is somewhat disturbed by impurities of the olive oil. However, as shown in Table 3, DLS-measurements showed particle sizes comparable to those found in the triolein-system which could be an indication for the formation of a comparable system even though it could not be confirmed by SAXS.

## 4. Discussion

In this contribution, we have shown the successful production of various types of inverse ISAsomes in two different systems. The first system used squalane as oil matrix and hydrophilic lyotropic LCs made of Genapol LA 070 (primary surfactant) and H_2_O were dispersed and stabilised with the help of hydrophobised silica nanoparticles (Pickering stabilisation). Apart from inverse hexosomes, inverse micellar cubosomes and an inverse emulsified microemulsion (EME) were also found at different concentrations of the primary surfactant. To the best of our knowledge, this is the first report on the two latter types of inverse ISAsomes. The confined hydrophilic structures showed the capability to incorporate finite amounts of oil until saturation without deteriorating. The Pickering-stabilised dispersion droplets showed fast aggregation (aggregate size of (100–300) µm) but no phase separation could be observed in a time span of one month. Similar to previous contributions [130], the interaction of the stabiliser led to a decrease in the effective surfactant concentration δ. The primary surfactant tends to adsorb to the stabiliser until saturation is reached which requires larger amounts of the primary surfactant to achieve the structures that were previously observed in oil-saturated bulk samples. Stabilisation attempts with molecular stabilisers were not successful, and the future replacement of the silica nanoparticles with a food-grade stabiliser could enable a wider range of possible applications.

The second system used triolein as the oil-continuous phase. Particles made of P94 (primary surfactant) and H_2_O were dispersed and stabilised with the molecular stabiliser Cithrol DPHS. Inverse hexosomes and an emulsified *L*_1_-phase were found. In contrast to an inverse EME, no oil is incorporated into its micellar nanostructure. The droplet size was in the range of (100–300) nm and no signs of phase separation could be found over the course of a month. In addition, stabiliser-free inverse hexosomes with aggregate sizes of (100–300) µm were produced. Most likely, the long polymeric chains of the primary surfactant lead to a steric stabilising effect that is sufficiently strong to prevent phase separation. The particles were named “self-stabilising inverse hexosomes” and could be beneficial in, e.g., skin care applications where the presence of a large amount of surfactant molecules is often undesired. In all cases, lyotropic LC structures made of P94/H_2_O incorporated hardly any triolein, a result that could also be replicated with other types of oil. As triolein is a highly pure model oil for natural plant oil, the replacement of triolein with commercially available olive oil was attempted. This resulted in the successful production of inverse hexosomes, both sterically stabilised and self-stabilising ones, with droplet sizes comparable to the twin system in triolein. To the best of our knowledge, this is the first report on inverse hexosomes formulated with a commercially available plant oil. The tendency of the binary system P94/H_2_O to incorporate hardly any oil into lyotropic LC structures might be a hint at its capability to form inverse ISAsomes in a large variety of oils as the LC structures are mostly undisturbed by the presence of the oil phase.

When compared to “conventional” ISAsomes, inverse ISAsomes are a rather new field of research. The development of such systems in a variety of biocompatible oils could lead to many applications in the fields of pharmaceutical and food industry. Ointments or other oil-continuous substances could be formulated to contain inverse ISAsomes to enable the controlled release of medical agents after being absorbed by the skin. The self-stabilising system presented in this contribution could be of special interest here due to the absence of a stabilising secondary surfactant. In addition, inverse ISAsomes could be used for the encapsulation of nutrients and enrichment of food oils. Combined with the ability to build in a controllable release trigger for these nutrients, the nutritional value of various oils could be enhanced. The presented formulations in triolein and olive oil are a first step towards the formulation of fully food-grade inverse ISAsomes. In addition to the replacement of the highly pure model oil triolein with a larger variety of natural food oils, future research could focus on the optimisation of the other ingredients. As many poloxamers such as P94 are approved as food additives, future studies could focus on the replacement of the molecular stabiliser with a suitable molecular or Pickering stabiliser. Various other possibilities such as the incorporation of active molecules (e.g., enzymes) into the inverse ISAsomes could be explored and the formulation of bicontinuous inverse cubosomes remains untackled up to this day.

## 5. Conclusions

We have achieved, for the first time, the production of various types of inverse ISAsomes in bio-compatible oils. This contribution adds inverse micellar cubosomes with Fd3m symmetry, an inverse emulsified microemulsion, an emulsified *L*_1_-phase and self-stabilising inverse hexosomes to the ISAsome family. While formulations in excess squalane showed various kinds of LC-nanostructures, the successful production of inverse ISAsomes in the food-grade oil phases triolein and olive oil might be an especially interesting starting point for studies that could explore a fully food-grade system for the oral delivery of various kinds of nutrients. As the field of research on inverse ISAsomes is rather unexplored when compared to the well-established “conventional” ISAsome dispersions in aqueous solutions, we believe that this contribution can be a starting point for opening up new pathways for both, basic research and applications. Further studies of the given systems could include their characterisation with Cryo-TEM and other experimental techniques that were not within the scope of this project.

## Figures and Tables

**Table 1:** Overview of the LC-phases observed in bulk samples in the system Genapol/squalane (Gena/Squal). Samples with selected surfactant concentrations δ=20,40,60 are shown. The corresponding oil-saturated systems with the observed LC-phases are also shown. The lattice parameter a, where applicable, is also displayed. The sketches of the LC-phases correspond to the structure that was observed in the oil-saturated samples at each value of δ.

System Composition	*δ*	LC-Phase		*a*/nm
Gena	20	$L_{1}$	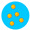	n.a.
Gena+Squal_20%_	20	$L_{1}$		n.a.
Gena	40	$H_{1}$	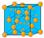	7.19
Gena+Squal_25%_	40	$I_{1}$		31.1
Gena	60	$H_{1}$	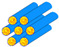	6.21
Gena+Squal_20%_	60	$H_{1}$		8.81

**Table 2:** Overview of the LC-phases observed in bulk samples in the system P94/Triolein. Samples with selected surfactant concentrations δ=30 and 55 are shown. The corresponding oil-saturated systems with the observed LC-phases are also shown. The lattice parameter a of the hexagonal phase is also displayed. The sketches of the LC-phases correspond to the structure that was observed in the oil-saturated samples at each value of δ.

System Composition	*δ*	LC-Phase		*a*/nm
P94	30	$L_{1}$	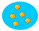	n.a.
P94+Triolein_5%_	30	$L_{1}$		n.a.
P94	55	$H_{1}$	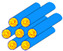	10.93
P94+Triolein_20%_	55	$H_{1}$		10.93

**Table 3:** DLS results for inverse ISAsomes dispersed in triolein and olive oil at various surfactant concentrations δ with a fixed stabiliser concentration β=5 and a fixed ISAsome concentration ϕ=10. The mean diameter *d* as well as the polydispersity index (*PDI*) are shown.

Oil Phase	*δ*	*d*/nm	*PDI*
Triolein	30	216	23%
Triolein	60	134	23%
Olive oil	30	175	37%

## Data Availability

Measurement data available upon request to the corresponding author.
